# Serological evidence of *Flavivirus* circulation in human populations in Northern Kenya: an assessment of disease risk 2016–2017

**DOI:** 10.1186/s12985-019-1176-y

**Published:** 2019-05-17

**Authors:** E. Chepkorir, D. P. Tchouassi, S. L. Konongoi, J. Lutomiah, C. Tigoi, Z. Irura, F. Eyase, M. Venter, R. Sang

**Affiliations:** 1 International Centre of Insect Physiology and Ecology, P. O. Box 30772-00100, Nairobi, Kenya; 2 Center for Viral Zoonoses, Department of Medical Virology, University of Pretoria, P. O. Box 323, Arcadia, 0007 South Africa; 3 Center for Virus Research, Kenya Medical Research Institute, P. O. Box 54628-00200, Nairobi, Kenya; 4 Division of Disease Surveillance and Response, Ministry of Health, P. O. Box 20781-00202, Nairobi, Kenya; 5 Jomo Kenyatta University of Agriculture and Technology, P.O. Box 606, Village Market, Nairobi, Kenya

**Keywords:** Dengue virus, Yellow fever virus, West Nile virus, Zika virus, Seroprevalence, Plaque reduction neutralization test, *Flaviviruses* risk assessment, Northern Kenya

## Abstract

**Background:**

Yellow fever, Dengue, West Nile and Zika viruses are re-emerging mosquito-borne *Flaviviruses* of public health concern. However, the extent of human exposure to these viruses and associated disease burden in Kenya and Africa at large remains unknown. We assessed the seroprevalence of Yellow fever and other *Flaviviruses* in human populations in West Pokot and Turkana Counties of Kenya. These areas border Uganda, South Sudan and Ethiopia where recent outbreaks of Yellow fever and Dengue have been reported, with possibility of spillover to Kenya.

**Methodology:**

Human serum samples collected through a cross-sectional survey in West Pokot and Turkana Counties were screened for neutralizing antibodies to Yellow fever, Dengue-2, West Nile and Zika virus using the Plaque Reduction Neutralization Test (PRNT). Seroprevalence was compared by county, site and important human demographic characteristics. Adjusted odds ratios (aOR) were estimated using Firth logistic regression model.

**Results:**

Of 877 samples tested, 127 neutralized with at least one of the four flaviviruses (14.5, 95% CI 12.3–17.0%), with a higher proportion in Turkana (21.1%, *n* = 87/413) than in West Pokot (8.6%, *n* = 40/464). Zika virus seroprevalence was significantly higher in West Pokot (7.11%) than in Turkana County (0.24%; χ^2^ *P* < 0.0001). A significantly higher Yellow fever virus seroprevalence was also observed in Turkana (10.7%) compared to West Pokot (1.29%; χ^2^ P < 0.0001). A high prevalence of West Nile virus was detected in Turkana County only (10.2%) while Dengue was only detected in one sample, from West Pokot. The odds of infection with West Nile virus was significantly higher in males than in females (aOR = 2.55, 95% CI 1.22–5.34). Similarly, the risk of Zika virus infection in West Pokot was twice higher in males than females (aOR = 2.01, 95% CI 0.91–4.41).

**Conclusion:**

Evidence of neutralizing antibodies to West Nile and Zika viruses indicates that they have been circulating undetected in human populations in these areas. While the observed Yellow Fever prevalence in Turkana and West Pokot Counties may imply virus activity, we speculate that this could also be as a result of vaccination following the Yellow Fever outbreak in the Omo river valley, South Sudan and Uganda across the border.

**Electronic supplementary material:**

The online version of this article (10.1186/s12985-019-1176-y) contains supplementary material, which is available to authorized users.

## Background

Yellow fever virus (YFV), Dengue virus (DENV), West Nile virus (WNV) and Zika virus (ZIKV) are important mosquito-borne *Flaviviruses* that have a potential to cause severe disease and mortalities in humans with economic and ecological consequence [1, 2]. Approximately 831 million people in Africa are at risk of infection with at least one of these viruses [3]. Epidemiologic studies on these mosquito-borne viruses remain a priority given their risk of global spread and high epidemic potential.

There have been no cases of Yellow Fever (YF) in Kenya since the first and last ever documented outbreak in 1992–95 [4, 5]. However, recent re-emergence of the virus with major outbreaks in border countries of Uganda, Ethiopia and South Sudan [6–9] and regionally in Angola and Democratic Republic of Congo has become a major public health concern [10]. Dengue is currently endemic in parts of Kenya, with outbreaks reported in some specific geographic zones in the coast and northern frontier of Kenya associated with dengue Serotypes 1–3, and neighboring countries like South Sudan, Somalia and Tanzania [11, 12]. West Nile on the other hand, was first isolated in Uganda in 1937 and is now one of the re-emerging zoonotic mosquito-borne pathogens whose occurrence and geographical range continues to spread [2].

Zika virus was first discovered in Uganda in 1947 [13], and since then, there has been limited data available on its circulation in the region. Previously, virological and immunological evidence suggested that although ZIKV was distributed widely in Africa and Asia, Zika fever was not a disease of substantial concern to human beings because only 14 cases had been documented worldwide [14, 15]. In 2016 outbreaks of the virus were reported in the Americas [16] that led to the World Health Organization (WHO) declaration of Zika virus as a public health emergency of international concern. The increased detection of Zika virus worldwide and its association with increasingly large outbreaks of disease has heightened awareness of this emerging mosquito-transmitted pathogen [17].

There is limited epidemiologic knowledge about the presence and spread of arboviral diseases in northern Kenya. Surveillance capacities are lacking, with most resources for study and control of these viruses being focused on epidemic periods. As such, the magnitude of human exposure and the burden of these important *Flavivirus* diseases in this region remain poorly understood. West Pokot and Turkana Counties are located in areas that border countries that have had and reported outbreaks of these *Flaviviruses* in recent times and therefore the risk of virus spread from neighboring countries remains high. This study sought to determine the seroprevalence of Yellow fever, Dengue, West Nile and Zika viruses among the human populations in the border locations of West Pokot County bordering Uganda and Turkana County bordering South Sudan and Ethiopia to the north.

## Materials and methods

### Ethical approval

This study was approved by Kenya Medical Research Institute (KEMRI) Scientific and Ethics Review Unit (SERU) (under protocol number KEMRI-SERU 2787) and University of Pretoria. All adult participants provided written informed consent and assent for child/minor participants. Only those who consented or assented were included in the study.

### Study sites and study population

**Fig. 1:**
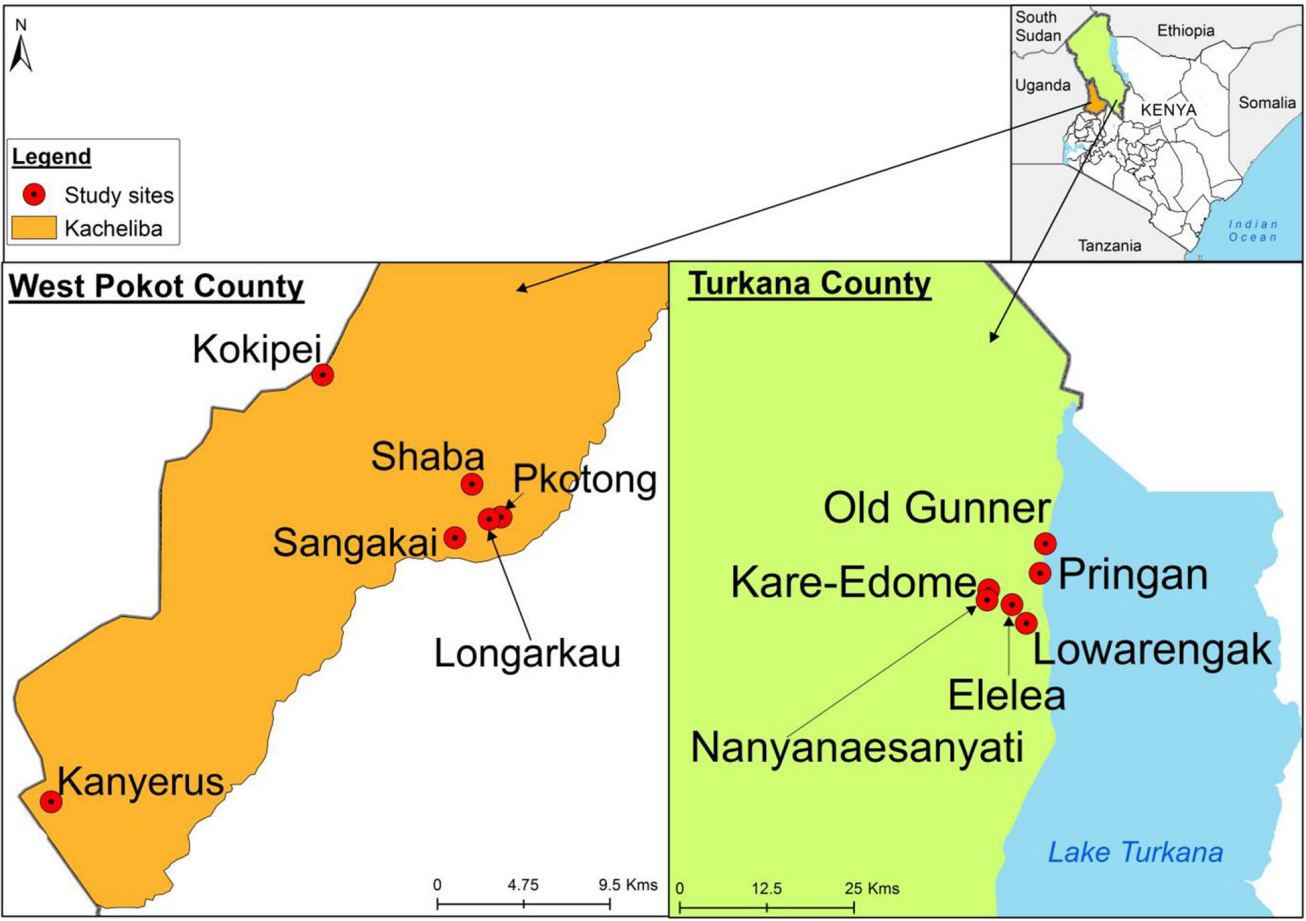
Map of Kenya showing the study sites in West Pokot and Turkana Counties. This map was generated specifically for this manuscript by *icipe* GIS team and does not infringe on copyright issues. The sample points were collected using a GPS gadget (garmin etrex 20, https://buy.garmin.com/en-US/US/p/518046), and the county boundaries for Kenya derived from AfricaOpendata (https://africaopendata.org/dataset/kenya-counties-shapefile, license Creative Commons)

This was a cross sectional population based study conducted in Turkana and West Pokot Counties of Kenya. Six sites each were selected and sampled in West Pokot County: Kacheliba sub-county (Kokipei, Sangakai, Kanyerus, Shaba, Longarkau and Pkotong) and Turkana County, Lokitoung sub-county (Elelea, Prigan, Kare-Edome, Nayanaesanyati, Old gunner and Lowarengak) (Fig. 1). Turkana County borders three high risk countries for YF, based on past and recent outbreaks. These include Ethiopia to the North, along the Omo River Valley, which experienced one of the largest YF outbreaks in East Africa in 1968 [18] and recently in 2013 [9]. Also, to the North is South Sudan and Uganda to the west. West Pokot borders Uganda which experienced an outbreak of YF in 2011 and 2016 [7, 8]. All the three neighboring countries have experienced YF outbreaks in locations that are close to Turkana and Kacheliba (West Pokot; Fig. 1).

The human populations in West Pokot are mainly nomadic pastoralists although there is increasing tendency for them to move to market centers and become sedentary. In Turkana, they are mainly nomadic pastoralists and tend to look for pasture in areas surrounding the lake, and some engage in fishing. As of 2009, the population of Turkana was 855,399 and West Pokot 512,690 [19]. The livelihoods of these communities are characterized by lack of most basic amenities like water, health care, education and even food. Corollary, they sometimes migrate as far as the neighboring countries of Uganda, South Sudan and Ethiopia in search of water and pasture, a practice that may put them at risk of exposure to YF and other exotic viruses.

### Sample collection

Human blood samples were collected through a cross-sectional study designed into village clusters based on administrative boundaries, in West Pokot and Turkana Counties, Kenya (Fig. 1) in February 2016 and August 2017, respectively. Blood samples were collected from a randomized asymptomatic human population within the village clusters. Both males and females aged between 12 and 95, who consented/assented to participate in the study, were included. An individual was excluded if he/she declined to give assent/consent. Gender, age, place of residence, occupation, YF vaccination and travel history were recorded for each study participant. A total volume of 5 ml of blood was collected from each study participant using vacutainer tubes with a clot activator. The tubes with blood samples were left to stand at room temperature for 10 min and then placed on sample carrier at 2–8 °C for transport to the field laboratory. At the field laboratory, the serum was processed by centrifugation at a relative centrifugal force (RCF) of 112 for 3 min, then aliquoted into 1 ml volumes, placed in two sterile cryovials. All the serum samples collected were preserved in liquid nitrogen and transported to the Emerging infectious Disease (EID) laboratory at the International Centre of Insect Physiology and Ecology (*icipe*) Duduville Campus in Nairobi, where they were stored at − 80 °C until tested.

### Plaque reduction neutralization assay (PRNT)

Samples were retrieved from the freezer, aliquoted in volumes of 30 μl, heat inactivated at 56 °C for 30 min and then tested for neutralizing antibodies to YF (YFXSMB), Dengue-2 (008/01/2012), West Nile (AMH005348) and Zika (MR766) viruses in two-fold serum dilutions from 1:10 to 1:320 using PRNT_90_. Respective virus isolates were diluted to a standard concentration that gave 20–50 plaques. Appropriate 10-fold dilutions of serum samples were prepared in maintenance media (Minimum Essential Media Eagles, Sigma with Earle̕ s salts and reduced NaHCO_3_) supplemented with 2% heat-inactivated fetal bovine serum (FBS), 2% L-glutamine, 2% penicillin/amphotericin B (Sigma-Aldrich, St. Louis, MO), starting with a seropositivity threshold of 1:10. The serum dilutions were added to microcentrifuge tubes containing the standard concentration of the diluted virus and incubated for 1 h at 37 °C. The virus–antibody mixture was inoculated on a 24-well plate containing confluent Vero cell monolayer (VERO E6) and incubated for 1 h for virus adsorption. After adsorption process, an overlay of methylcellulose (2.5%) (Sigma) mixed with 2X Minimum Essential Medium (Sigma), was added in to the wells. Between 6 and 14 days depending on the virus tested, the plaques were fixed using 10% formalin (Sigma), stained with 0.25% crystal violet (Sigma) diluted in absolute ethanol and the plaques counted manually as described previously [20, 21]. Endpoint titres were determined as the reciprocal of the highest serum dilution giving ≥90% reduction in plaque counts [22].

### Statistical analysis

Field data captured were stored in a password-protected database linked to the laboratory results, and were analyzed using R version 3.3.1 [23]. First, we characterized the study participants with respect to exposure to the *Flaviviruses* tested. Reported prevalence was compared by county, site and important demographic characteristics such as sex, age, occupation and YF vaccination status. Comparisons of proportions and evaluation of heterogeneity of the seropositive proportions across the independent variables listed above were performed using Chi Square test. The 95% confidence intervals (CIs) for the proportions positive for a virus were estimated using the Agresti-Coull method [24]. Next, we fitted for each of the three most prevalent *Flaviviruses* with a frequency of six or more positive samples (i.e., YFV in both West Pokot and Turkana; ZIKV in West Pokot; WNV in Turkana), a multiple logistic regression model with covariates as site, sex, age in years, occupation, whether the participant was a herdsman, and whether the participant reported to have or have not received YF vaccination. As the data were sparse, Firth’s logistic regression [25] was chosen. Firth’s approach reduces the bias in maximum likelihood (ML) estimates of coefficients when binary events are rare by penalizing the likelihood function using Jeffreys invariant prior. In addition, it also allows for the computation of reliable, finite estimates of coefficients in the case of separation, where ML estimation fails [26]. All tests were performed at 5% significance level.

## Results

### Demographic profile of study participants

Table 1 shows the distribution of the study participants with respect to their demographic characteristics for the individual and combined counties. A total of 877 serum samples were collected; 464 from West Pokot and 413 from Turkana. Of these samples, 64% (565/877) were from females and 36% (312/877) males. In both counties, there were more female participants than males. The males were often away from the homesteads as their occupational activities (fishing and farming) kept them away at the time of enrolment and sampling. The sampled participants’ age ranged from 13 to 92 years, and their mean (median) age was 39 years (35 years). About three in five study participants were farmers, but this varied across the counties as almost all the participants (98.5%) in West Pokot were farmers. About 23% of the study participants were housewives or female domestic servants, 9% fishermen, 2.4% were either teachers or students, and the rest were involved in other economic activities. Most participants (42%) indicated they had not been vaccinated against YF, 36% did not know their YF vaccination status, and 22% indicated that they had been vaccinated.

**Table 1:** Demographic characteristics of study participants from Kacheliba in West Pokot and Lokitoung in Turkana Counties

Characteristic	West Pokot County		Turkana County		Combined	
	N	%	N	%	N	%
All	464	100.0	413	100.0	877	100.0
Sex						
Female	324	69.8	241	58.4	565	64.4
Male	140	30.2	172	41.6	312	35.6
Age (years)						
13–19	14	3	48	11.6	62	7.1
20–29	101	21.8	139	33.7	240	27.4
30–39	106	22.8	86	20.8	192	21.9
40–49	77	16.6	38	9.2	115	13.1
50+	166	35.8	102	24.7	268	30.6
Occupation						
Farmer	457	98.5	71	17.2	528	60.2
Wife/housegirl	0	0.0	198	47.9	198	22.6
Fisherman/vendor	0	0.0	82	19.9	82	9.4
Teacher/student	4	0.9	17	4.1	21	2.4
Other	3	0.7	45	10.9	48	5.5
Herdsman						
No	425	91.6	373	90.3	798	91.0
Yes	39	8.4	40	9.7	79	9.0
Yellow Fever vaccinated						
No	192	41.4	176	42.6	368	42.0
Yes	182	39.2	11	2.7	193	22.0
don’t know	90	19.4	226	54.7	316	36.0

### Prevalence of *Flaviviruses* in west Pokot and Turkana counties

**Fig. 2:**
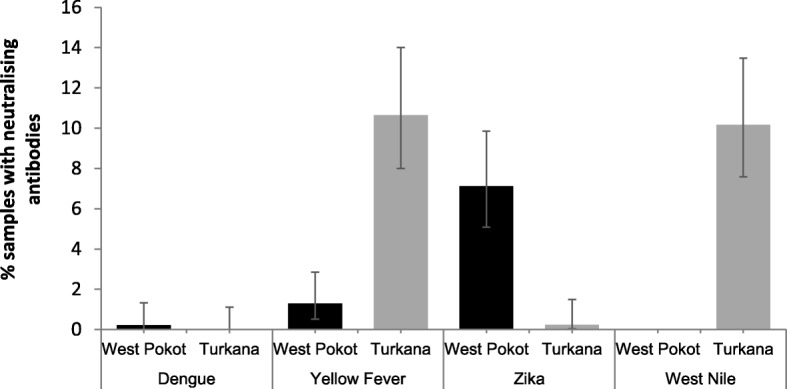
Seroprevalence of the various *Flaviviruses* presented separately for each county. The error bars indicate Agresti-Coull 95% confidence intervals. The number of samples tested was 413 in Turkana and 464 in West Pokot

Of 877 samples tested, 127 (14.5, 95% CI 12.3–17.0%) were positive for at least one of the four *Flaviviruses*. This proportion was, however, higher in Turkana (21.1%) than in West Pokot (8.6%; *P* < 0.0001). Figure 2 presents plaque reduction neutralization test results stratified by county. First, the figure shows that in general, the virus to which the most neutralizing antibodies were present was YFV, followed by WNV, ZIKV and DENV. Second, there was variability in the prevalence of the *Flaviviruses* between the two counties. Whereas DENV neutralizing antibodies were detected in one sample (0.22% prevalence) from West Pokot only, WNV antibodies were detected in Turkana only (42/413, prevalence = 10.2%). While YFV and ZIKV antibodies were detected in samples from both counties, YFV was significantly more prevalent in Turkana (10.7%) than West Pokot (1.29%; χ^2^ P < 0.0001). The converse was true for ZIKV.

Table 2 summarizes PRNT results across six sites within each county. There was variability in *Flavivirus* prevalence across the sites within as well as between the counties. Yellow fever prevalence ranged from 0% in Kokipei, Shaba, Longarkau, and Pkotong to about 4% in Kanyerus in West Pokot. In Turkana, YF prevalence varied from about 7% in Kare Edome to about 20% in Elelea. For West Nile virus antibodies, which were detected in Turkana only, the prevalence varied from 8% in Prigan to 11.1% in Elelea. Also samples from only two sites had neutralizing antibodies to three viruses detected. These were Sangakai in West Pokot (DEN, YF, and ZIK) and Elelea in Turkana (YF, ZIK, and WN).

**Table 2:** *Flavivirus* PRNT_90_ endpoint titre results for sites in Kacheliba, West Pokot County and Lokitoung, Turkana County

Site	Number of samples tested	Number of samples (%) positive of each Flavivirus			
		Dengue	Yellow Fever	Zika	West Nile
West Pokot					
Kokipei	100	0 (0.00)	0 (0.00)	8 (8.00)	0 (0.00)
Sangakai	66	1 (1.52)	2 (3.03)	6 (9.09)	0 (0.00)
Kanyerus	113	0 (0.00)	4 (3.54)	13 (11.50)	0 (0.00)
Shaba	73	0 (0.00)	0 (0.00)	3 (4.11)	0 (0.00)
Longarkau	60	0 (0.00)	0 (0.00)	1 (1.67)	0 (0.00)
Pkotong	52	0 (0.00)	0 (0.00)	2 (3.85)	0 (0.00)
Total	464	1 (0.22)	6 (1.29)	33 (7.11)	0 (0.00)
Turkana					
Elelea	63	0 (0.00)	12 (19.05)	1 (1.59)	7 (11.11)
Prigan	25	0 (0.00)	2 (8.00)	0 (0.00)	2 (8.00)
Kare Edome	61	0 (0.00)	4 (6.56)	0 (0.00)	6 (9.84)
Nayanaesanyati	69	0 (0.00)	6 (8.70)	0 (0.00)	7 (10.14)
Old gunner	56	0 (0.00)	5 (8.93)	0 (0.00)	5 (8.93)
Lowarengak	139	0 (0.00)	15 (10.79)	0 (0.00)	15 (10.79)
Total	413	0 (0.00)	44 (10.65)	1 (0.24)	42 (10.17)

**Fig. 3:**
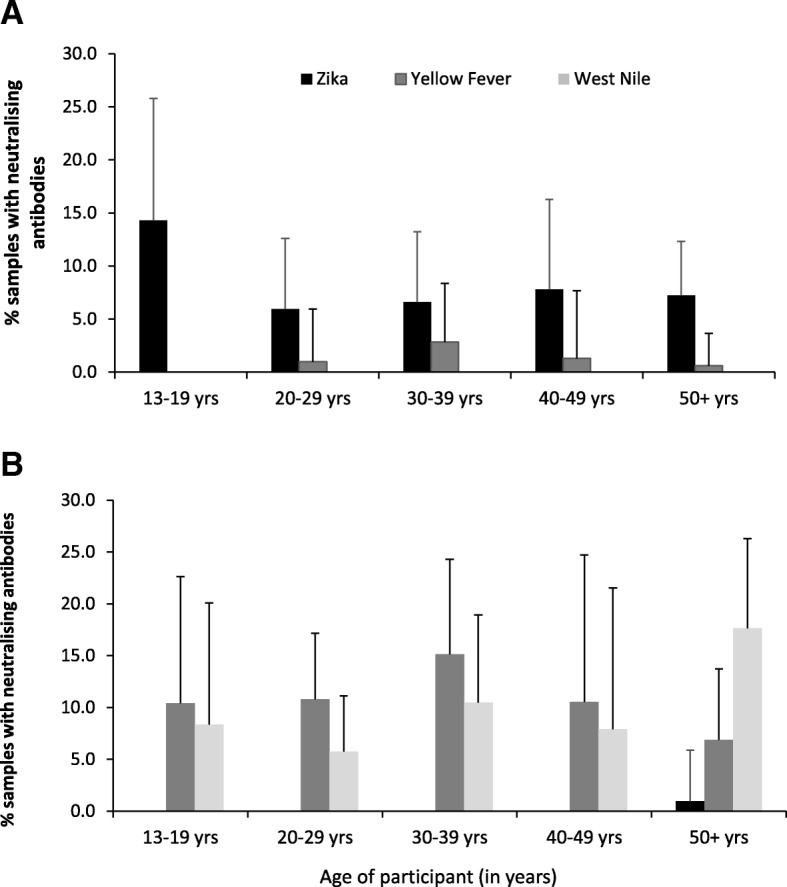
Proportions positive for three most prevalent viruses (ZIK, YF and WN) by age group for (**a**) West Pokot (*n* = 464) and (**b**) Turkana (*n* = 413) counties. The error bars indicate Agresti-Coull 95% confidence intervals

Prevalence of the three most prevalent *Flaviviruses* (ZIKV, YFV and WNV) varied by age group in both counties (Fig. 3). In West Pokot, the prevalence of ZIKV was highest among those in the age group 13–19 years and lowest among the age group 20–29 years, though the difference was not significant. Yellow fever neutralizing antibodies were more among the age group 30–39 years and less among those aged 50+ years, though the difference was not significant. In Turkana County, the prevalence of WNV was highest among those aged ≥50 years (χ^2^ *P* = 0.048) and lowest among the age group 20–29 years. Zika virus antibodies were only detected in the 50+ years age group in Turkana County. In general, variation between seroprevalence to *Flaviviruses* was quite limited between age groups.

*Flaviviruses* that had more than 30 samples neutralizing with either of the three most prevalent viruses for each county (i.e., West Pokot: ZIKV (*n* = 33); Turkana: YFV (*n* = 44), WNV (*n* = 42) by demographic characteristics are presented in Additional file 1: Table S1.

### Firth’s logistic regression model results

Table 3 shows the adjusted odds ratios (aORs) and the associated 95% confidence intervals estimated using Firth’s logistic regression model. The data show that in Turkana County, males were about three times more likely to be infected with WNV than females (aOR = 2.55, 95% CI 1.22–5.34). There was a two-fold likelihood of WNV infection among the age group 50+ years compared to the age group 13–19 years. There was no difference in YF seroprevalence in Turkana by site, sex, age group and vaccination status. In West Pokot County, YF infection was eight times higher among study participants from Sangakai (aOR = 8.24, 95% CI 0.31–220.15) and Kanyerus (aOR = 8.32, 95% CI 0.40–172.04), and two times highly likely in participants from Longarkau (aOR = 2.02, 95% CI 0.04–110.05) and Pkotong (aOR = 2.02, 95% CI 0.04–110.5). The odds of infection with ZIKV in West Pokot was twice as high in males compared to females (aOR = 2.01, 95% CI 0.91–4.41).

**Table 3:** Comparison of *Flavivirus* prevalence by site, demographic parameters and history of YF vaccination from Firth’s multiple logistic regression model

Variable	West Pokot (*N* = 464)				Variable	Turkana (*N* = 413)			
	Yellow fever (*n* = 6)		Zika (*n* = 33)			Yellow Fever (*n* = 44)		West Nile (*n* = 42)	
	aOR (95% CI)	*P*value	aOR (95% CI)	*P*value		aOR (95% CI)	*P*value	aOR (95% CI)	*P*value
Site					Site				
Kokipei	Reference		Reference		Elelea	Reference		Reference	
Sangakai	8.24 (0.31–220.15)	0.208	0.77 (0.23–2.60)	0.669	Prigan	0.42 (0.10–1.79)	0.240	0.98 (0.21–4.69)	0.982
Kanyerus	8.32 (0.40–172.04)	0.170	1.10 (0.41–2.93)	0.853	Kare Edome	0.32 (0.10–1.04)	0.058	0.94 (0.28–3.17)	0.925
Shaba	1.46 (0.03–77.85)	0.851	0.45 (0.12–1.70)	0.241	Nayanaesanyati	0.36 (0.12–1.07)	0.066	1.21 (0.37–3.92)	0.749
Longarkau	2.02 (0.04–110.5)	0.731	0.24 (0.04–1.46)	0.122	Old gunner	0.39 (0.12–1.22)	0.106	0.88 (0.24–3.14)	0.838
Pkotong	2.02 (0.04–104.34)	0.728	0.36 (0.08–1.67)	0.191	Lowarengak	0.39 (0.15–0.99)	0.047	1.45 (0.49–4.31)	0.505
Sex					Sex				
female	Reference		Reference		female	Reference		Reference	
male	0.40 (0.04–4.62)	0.466	2.01 (0.91–4.41)	0.084	male	0.84 (0.41–1.72)	0.631	2.55 (1.22–5.34)	0.013
Age group					Age group				
13–19	Reference		Reference		13–19	Reference		Reference	
20–29	0.82 (0.03–24.26)	0.910	0.35 (0.07–1.81)	0.209	20–29	1.26 (0.41–3.84)	0.686	0.76 (0.22–2.66)	0.663
30–39	1.76 (0.08–38.73)	0.721	0.42 (0.09–2.07)	0.288	30–39	1.59 (0.52–4.84)	0.414	1.43 (0.41–4.92)	0.574
40–49	1.06 (0.04–30.49)	0.975	0.45 (0.09–2.31)	0.337	40–49	1.03 (0.26–4.01)	0.970	1.09 (0.24–4.94)	0.912
50+	0.39 (0.01–12.26)	0.595	0.34 (0.07–1.59)	0.169	50+	0.72 (0.22–2.38)	0.589	2.10 (0.66–6.66)	0.206
Herdsman					Herdsman				
No	Reference		Reference		No	Reference		Reference	
Yes	3.16 (0.2–48.99)	0.411	1.16 (0.36–3.72)	0.806	Yes	0.93 (0.27–3.28)	0.915	1.40 (0.52–3.74)	0.508
Yellow fever vaccinated					Yellow fever vaccinated				
No	Reference		Reference		No	Reference		Reference	
Yes	0.61 (0.09–3.91)	0.599	1.05 (0.42–2.60)	0.924	Yes	0.81 (0.13–5.13)	0.819	1.70 (0.34–8.57)	0.519
Don’t know	1.45 (0.22–9.79)	0.701	2.90 (1.11–7.60)	0.030	Don’t know	1.21 (0.61–2.41)	0.586	0.75 (0.37–1.53)	0.427

## Discussion

The present study reports on the serological evidence of human exposure to *Flaviviruses* in West Pokot and Turkana counties of northern Kenya. Overall, we found that 14.5% (*n* = 127, 95% CI 12.3–17.0%) of the samples were positive for at least one flavivirus (ZIKV, YFV, DENV-2, WNV). However, there were variations in the rates of human exposure for the individual viruses between the counties and across sites. Such variation in prevalence by site or even region is not uncommon in Kenya [27–29]. At the county level, seroprevalence ranged from 0.22% for DENV in West Pokot to 10.7% for YFV in Turkana. Much higher exposure of specific flavivirus has been observed, of up to 60% for DENV in coastal Kenya [27, 30]. Such variability in specific flavivirus as observed in our study and elsewhere in Kenya suggests exposure is likely conditioned by a myriad of ecological and human factors such as vector species and abundance, climate and weather patterns, environmental features, occupational activities, degree of mobility among others.

West Nile virus was detected in Turkana (42/413, 10.2%), but not in West Pokot. This difference could relate partly to ecological variation between the two areas. West Nile virus is associated with *Culex* mosquitoes, the virus having been previously isolated in some *Culex* species in Kenya [31–33]. It is possible the mosquito fauna especially the *Culex* spp. abundance and diversity varies between the areas. Grossi-Soyster [29] found high risk of both alphaviruses and flaviviruses primarily associated with households near the Lake area in Western Kenya. In this case, the presence of a lake (Lake Turkana) may encourage high diversity of bird species and breeding of *Culex* species, which are important determinants of the epidemiology of WNV [2]. This may possibly account for higher exposure rates in humans in Turkana than West Pokot. Proximity to Lake Turkana may also influence the economic activities of the people living in the area, such as fishing or grazing livestock. Older men spend more time away from home fishing, taking care of the livestock and looking for pasture in areas around the lake. Such outdoor activities could predispose them to more mosquito infectious bites. This could explain the obvious disparity in exposure rates among males compared to females, even though more samples were analyzed from the latter relative to the former. In fact, males were about three times more likely to be infected with WNV than females (aOR = 2.55, 95% CI 1.22–5.34). Additionally, adult males aged 50+ had more exposure, consistent with previous findings [30]. This alludes to higher risk due to advanced age; however, exposure in the 13–19 age group is indicative of ongoing transmission of this virus. Evidence of isolation of the virus from *Culex* spp. in this ecology [33] lends support for active virus transmission.

Neutralizing antibodies against YFV were detected in samples from both counties, although significantly higher in Turkana than West Pokot (*P* < 0.0001). Site specific variation in exposure rates was also evident ranging from 7% in Kare Edome to about 20% in Elelea in Turkana County. It has been about three decades since the last documented outbreak of YF in Kenya in the neighboring Rift Valley [4, 5]. The exposure in the 13–19 age group, although higher in 30–39 age category, could indicate low-level ongoing transmission and persistent exposure in this region. The participants’ responses to their YF vaccination status made it difficult to determine the definite source of infection for YF reactive samples. Record of vaccination status was based on self-reporting which could not be verified, as no vaccination cards were provided during survey. However, the presence of YF neutralizing antibodies in these two counties may be attributable in part to the vaccinations that were rolled out in 2003 in response to the outbreak that was reported in the neighboring South Sudan [6]. Additionally, anecdotal information from the Kenyan Ministry of Health and Sanitation suggests that, some of the population received YF vaccination in Uganda in response to the 2011 YF outbreak [7], while they had crossed the borders to Uganda for pasture and trade. However, the level of immunity seems too low for a population that was vaccinated so recently to forestall an outbreak from a neighboring country. If vaccination was carried out to prevent an outbreak, then a seroprevalence of 10% is a far cry from the desired protective herd immunity level (80%) and should be enhanced. We also cannot rule out the circulation of YF in low levels in these regions, because of cross border travel by this population to outbreak areas in the neighboring countries in search of pasture and trade contributing to this high seroprevalence. Our findings somehow point to active circulation of YFV in low levels in these regions; similar exposure rates have been observed in other regions of Kenya [30].

Only one sample in West Pokot was reactive to DENV-2 and none from Turkana. This in part may suggest low human exposure to this serotype in this region. Previous studies have highlighted the prevalence of DEN-2 serotype in Kenya through serological studies [12, 28, 34]. Nonetheless, human exposure to the other serotypes (1, 3–4) has been reported [30]. While we employed PRNT which is the gold standard for determining serological specific among viruses, cross reactivity of human sera, however, occurs with all four DENV serotypes [35] and even with other flaviviruses. The limitation of our analysis to only a single serotype (DEN-2), suggests more studies are needed to determine the exact burden of DEN in this region and possibly establish the serotype distribution.

The results in the current study suggest circulation of Zika in both counties, yet there have never been reported or confirmed cases of ZIKV in Kenya. The prevalence of Zika was significantly higher in West Pokot than in Turkana County. Coincidentally, the highest seroprevalence was recorded in Kanyerus (Fig. 1), proximal to the Uganda where the virus was first detected in 1947 [13]. Exposure could be influenced by the degree of human mobility between this site and across border into Uganda. As nomadic pastoralists, long distance movement of livestock in search of pasture or water is common practice especially during drought. Few studies have reported data on human exposure to ZIKV in Kenya, the last dating > 40 years [36]. The high prevalence among the age group 13–19 years possibly indicates evidence of active circulation of this virus in this ecology, which may have gone unnoticed due to lack of detection facilities. This calls for focused arboviral surveillance to forestall severe consequences of the virus emergence and incriminate the important vectors to enhance preparedness in dealing with potential outbreaks.

Our sampling approach focused only on asymptomatic population and could not tell whether the exposure represented here did manifest clinically. It is well known that clinical presentations of arboviral diseases are often highly non-specific, commonly indistinguishable from each other and from other common tropical diseases like malaria [37]. The lack of diagnostic capability for arboviruses being routinely implemented in any health facility in the study area makes tracking of clinical cases very difficult, outside the confines of research studies. The need for continuous monitoring remains a priority given the unanticipated emergence or reemergence of arboviral diseases in recent years mainly in the form of outbreaks. Active surveillance including virus activity in vectors would help to shed light on the specific vectors involved and risk trajectory. Such studies could employ emerging technologies such as Next Generation Sequencing (NGS) to identify and characterize even unknown pathogens that could potentially be of human health impact.

This study has some limitations that should be considered. Our sampling approach targeted asymptomatic population in a cross-sectional study design. There was no clinical information related to these infections in the study population. Further studies should include clinical disease monitoring and should be extended to include young children and pregnant women, as in the case of ZIKV which has been found to be associated with congenital birth defects, including microcephaly in the developing fetus [38]. In PRNT analysis, we employed only DEN-2 serotype which affects definite conclusions that can be drawn regarding DEN exposure and other serotypes in the study areas. Also, while we used the Firth’s logistic regression model to reduce bias in estimates and to overcome the computational problems experienced with the conventional logistic regression model, the low frequencies of the viruses – as has been reported in the literature – does make it difficult to associate the prevalence with the risk factors.

## Conclusion

The findings have demonstrated human exposure to infections with *Flaviviruses* in West Pokot and Turkana counties of Kenya. The presence of West Nile and Zika virus neutralizing antibodies in this population especially in the 13–19 age group shows that the viruses have recently been circulating undetected in human populations in this region. This suggests that there is a risk of WNV transmission in Turkana and ZIKV transmission in West Pokot County. The variable exposure risk observed between the areas could be related to differences in climate and geography between them. Therefore, it would be worthwhile for future studies to assess the local mosquito fauna composition and abundance trends, and investigate their vector competence to transmit ZIKV, in order to shed light on the high prevalence observed. Evidence of YF seropositivity may partly be due to previous vaccination, and if so, there is a potential risk of YF transmission if vaccination is not enhanced to improve herd immunity to protective level of 80%, considering proximity to an outbreak area across the border. One Health approach through active surveillance to integrate the myriad of factors involved in modulating human exposure risk is crucial to develop a decision support tool for accurate risk assessment. Overall, the findings provide needed evidence of human exposure, for action towards disease prevention plans and the activation of preparedness strategies to counter potential threats posed by these viruses.

## Additional file

Additional file 1: **Table S1.** Prevalence of Zika virus (West Pokot County) and Yellow Fever, West Nile (Turkana County) by demographic characteristics. (DOCX 19 kb)

## Abbreviations

aORs: Adjusted odds ratios
CI: Confidence Interval
DENV: Dengue virus
EID: Emerging Infectious Diseases
GIS: Geographic Information System
GPS: Global Positioning System
ML: Maximum Likelihood
NGS: Next Generation Sequencing
PRNT: Plaque Reduction Neutralization Test
RPM: Revolutions per Minute
WHO: World Health Organization
WNV: West Nile virus
YFV: Yellow fever virus
ZIKV: Zika virus
