# Supplementary material for: Serological evidence of Flavivirus circulation in human populations in Northern Kenya: an assessment of disease risk 2016–2017
Source: Virol J. 2019 May 17;16:65. doi: 10.1186/s12985-019-1176-y (PMC6525424; doi:10.1186/s12985-019-1176-y)
Supplement: Supplementary file 1 — Table S1. Prevalence of Zika virus (West Pokot County) and Yellow Fever, West Nile (Turkana County) by demographic characteristics. (DOCX 19 kb) [file 12985_2019_1176_MOESM1_ESM.docx]

# Supporting information

Additional file 1: **Table S1.** Prevalence of Zika virus (West Pokot County) and Yellow Fever, West Nile (Turkana County) by demographic characteristics.

| Characteristic | West Pokot | | | Turkana | | | | | | | |
| --- | --- | --- | --- | --- | --- | --- | --- | --- | --- | --- | --- |
|  | Zika | | χ^2^  Pvalue | Yellow fever | | χ^2^  Pvalue | West Nile | | χ^2^  Pvalue |  |  |
|  | Number positive | Percent Positive |  | Number positive | Percent Positive |  | Number positive | Percent Positive |  |  |  |
| All | 33 | 7.1 |  | 44 | 10.7 |  | 42 | 10.2 |  |  |  |
| Sex |  |  |  |  |  |  |  |  |  |  |  |
| female | 18 | 5.6 |  | 29 | 12.0 |  | 14 | 5.8 |  |  |  |
| male | 15 | 10.7 | 0.047 | 15 | 8.7 | 0.282 | 28 | 16.3 | 0.001 |  |  |
| Age (years) |  |  |  |  |  |  |  |  |  |  |  |
| 13-19 | 2 | 14.3 |  | 5 | 10.4 |  | 4 | 8.3 |  |  |  |
| 20-29 | 6 | 5.9 |  | 15 | 10.8 |  | 8 | 5.8 |  |  |  |
| 30-39 | 7 | 6.6 |  | 13 | 15.1 |  | 9 | 10.5 |  |  |  |
| 40-49 | 6 | 7.8 |  | 4 | 10.5 |  | 3 | 7.9 |  |  |  |
| 50+ | 12 | 7.2 | 0.844 | 7 | 6.9 | 0.502 | 18 | 17.6 | 0.048 |  |  |
| Occupation |  |  |  |  |  |  |  |  |  |  |  |
| Farmer | 33 | 7.2 |  | 8 | 11.3 |  | 12 | 16.9 |  |  |  |
| Wife/housegirl | 0 | 0.0 |  | 22 | 11.1 |  | 13 | 6.6 |  |  |  |
| Fisherman/vendor | 0 | 0.0 |  | 6 | 7.3 |  | 9 | 11.0 |  |  |  |
| Teacher/student | 0 | 0.0 |  | 4 | 23.5 |  | 1 | 5.9 |  |  |  |
| Other | 0 | 0.0 | 0.762 | 4 | 8.9 | 0.388 | 7 | 15.6 | 0.086 |  |  |
| Herdsman |  |  |  |  |  |  |  |  |  |  |  |
| no | 28 | 6.6 |  | 41 | 11.0 |  | 34 | 9.1 |  |  |  |
| yes | 5 | 12.8 | 0.147 | 3 | 7.5 | 0.496 | 8 | 20.0 | 0.030 |  |  |
| Yellow Fever vaccinated | |  |  |  |  |  |  |  |  |  |  |
| no | 12 | 6.3 |  | 18 | 10.2 |  | 19 | 10.8 |  |  |  |
| yes | 10 | 5.5 |  | 1 | 9.1 |  | 2 | 18.2 |  |  |  |
| don't know | 11 | 12.2 | 0.106 | 25 | 11.1 | 0.951 | 21 | 9.3 | 0.595 |  |  |
